# Environmental pollutants as modifiers of antimicrobial resistance risk across food systems: a One Health perspective

**DOI:** 10.3389/fpubh.2026.1918334

**Published:** 2026-09-23

**Authors:** Kingsley Excel Dunga, Ebinyo Rebecca Aseibai, Nsikak Godwin Etim, Matthew Chidozie Ogwu, Vivian Ibienebakabobo Promise, Esther Ugo Alum, Sylvester Chibueze Izah

**Affiliations:** 1 Department of Medical Laboratory Science, Kingsley Ozumba Mbadiwe University, Ogboko, Imo State, Nigeria; 2 Department of Microbiology, Federal University Otuoke, Otuoke, Bayelsa State, Nigeria; 3 Niger-Delta Institute for Emerging and Re-emerging Infectious Diseases, Federal University Otuoke, Yenagoa, Bayelsa State, Nigeria; 4 Goodnight Family Department of Sustainable Development, Appalachian State University, Boone, NC, United States; 5 Department of Public Health, Faculty of Health Sciences, Bayelsa Medical University, Yenagoa, Bayelsa State, Nigeria; 6 Department of Research and Publications, Kampala International University, Kampala, Uganda; 7 Departments of Community Medicine and Microbiology, Bayelsa Medical University, Yenagoa, Bayelsa State, Nigeria

**Keywords:** antimicrobial resistance, environmental pollutants, exposure assessment, food systems, integrated surveillance, One Health, source attribution

## Abstract

Antimicrobial resistance (AMR) in bacteria associated with food systems is commonly attributed to antimicrobial use in clinical medicine and animal production, yet food-production and processing environments are also exposed to metals, biocides, pesticides, pharmaceutical residues, microplastics, sewage, and industrial effluents. These pressures can influence bacterial survival, biofilm persistence, antimicrobial susceptibility, and the maintenance or transfer of resistance determinants across soil, water, animals, crops, processing environments, markets, and households. This Perspective considers environmental pollutants as modifiers of AMR risk rather than sufficient evidence of causation or clinically relevant human transmission. Public-health concern is greatest where four elements converge: selective pollutant pressure, viable pathogens or clinically relevant colonizing or opportunistic organisms, mobile or expressed resistance determinants, and a credible human exposure pathway. This convergence model complements established One Health surveillance frameworks by explicitly linking environmental chemistry and pollutant mixtures with viability, resistance phenotype, mobile-element context, exposure, source attribution, and intervention evaluation. Evidence from antibiotic residues, metals, pesticides, biocides, microplastics, wastewater, genomic surveillance, and source-attribution studies indicates that biological plausibility alone is insufficient to establish human health risk. A pathway-based interpretation can distinguish environmental AMR signals from those supported by stronger evidence of food-system exposure and clinical relevance, while identifying surveillance and intervention priorities that can be tested across settings.

## 1 Introduction

Bacterial AMR remains a major global health threat. The 2024 Global Burden of Disease analysis estimated that 1.14 million deaths were attributable to bacterial AMR and 4.71 million deaths were associated with it in 2021 (1). Foodborne exposure is also substantial: the WHO comparative assessment estimated 600 million illnesses and 420,000 deaths from 31 foodborne hazards in 2010, with 40% of the disease burden occurring in children younger than 5 years (26). These figures are not estimates of foodborne AMR, but together they explain why resistant organisms moving through food systems deserve explicit public-health attention. AMR in food systems cannot be understood only by measuring antimicrobial use. Food-producing environments receive antibiotics and other pharmaceuticals, metals, disinfectants, pesticides, plastics, manure, sewage, and industrial waste. These exposures may select resistant variants, maintain genetically linked resistance determinants, alter permeability or efflux, encourage biofilms, or create ecological conditions that increase bacterial persistence and gene exchange (2–4). However, resistance also occurs naturally in environments with limited human influence, and multidrug resistance or detection of an ARG alone does not establish an anthropogenic foodborne threat (5).

One Health seeks to optimize the health of people, animals, plants, and ecosystems through integrated action (6). Integrated AMR surveillance is already well developed conceptually and operationally. WHO/AGISAR guidance links human, food-producing animal, retail-food, and other relevant surveillance components; the WHO Tricycle protocol provides a standardized low-resource model based on ESBL-producing *Escherichia coli* across human, food-chain, and environmental sectors; UNEP has strengthened the environmental dimension by identifying pollution sources, pathways, and prevention priorities; and the 2026 Quadripartite guidance provides objective-driven roadmaps for context-appropriate One Health integrated surveillance of AMR and antimicrobial use (7–10). The updated Global Action Plan on AMR 2026–2036 further reinforces coordinated One Health surveillance, prevention, environmental action, accountability, and measurable implementation (11). Against this background, a pollutant-explicit risk-prioritization layer can complement these frameworks by considering environmental chemistry is measured alongside organism viability, phenotypic resistance, resistance-gene mobility and expression, food exposure, source attribution, and clinical consequence. Three propositions follow: pollutants are modifiers of AMR risk rather than sufficient causal proof; priority hazards are combinations of viable organisms, mobile clinically relevant resistance, selective mixtures, and credible exposure; and surveillance should test transmission and intervention hypotheses rather than merely catalog pollutants, organisms, or genes. Figure 1 presents the convergence model and identifies the microbiological, chemical, genomic, exposure, and clinical evidence needed to test each proposed link (2–18).

**Figure 1:**
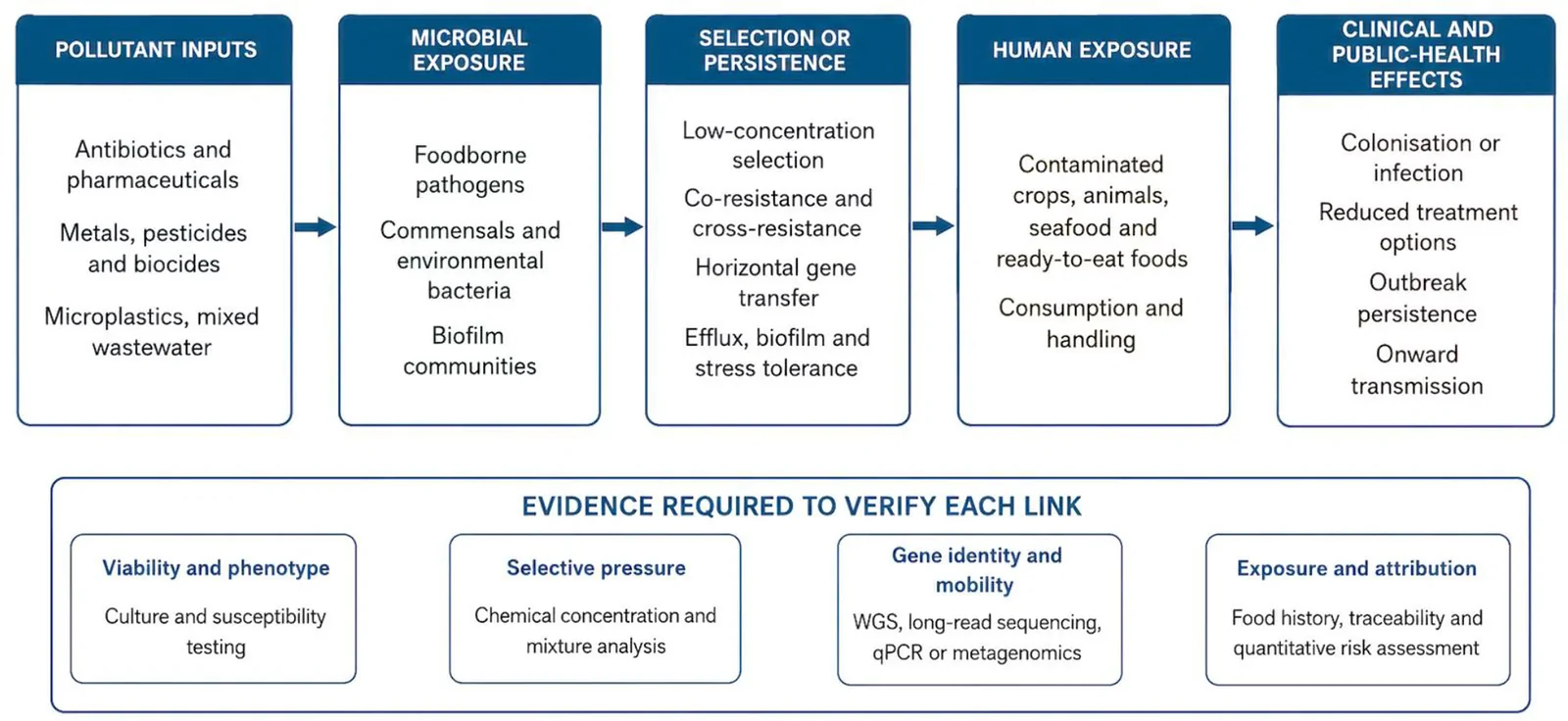
Convergence framework for pollutant-associated antimicrobial resistance risk in food systems. Pollutant inputs may influence microbial selection or persistence, but public-health relevance depends on evidence linking viable organisms or functionally relevant resistance determinants to human exposure and clinical outcomes. The lower panel identifies complementary microbiological, chemical, genomic, epidemiological, and risk-assessment evidence needed to evaluate each link. Source: conceptual synthesis based on (2–4, 7–10).

The discussion draws on peer-reviewed experimental, genomic, surveillance, and source-attribution studies together with international One Health guidance. Quantitative examples are used where they clarify a specific step between environmental exposure, microbial response, food-system transmission, and human relevance. They are interpreted as study-specific evidence rather than pooled effect estimates or globally representative prevalence values because pollutant concentrations, organisms, matrices, analytical methods, and production conditions differ substantially among settings. This approach is consistent with the purpose of a Perspective: to integrate established evidence, identify interpretive limitations, and define testable priorities for research, surveillance, and action.

## 2 Environmental pollutant pressures and evidence

### 2.1 From exposure to selection

Environmental pressures differ in persistence, bioavailability, concentration, and evidential strength. Table 1 summarizes major pollutant groups, AMR-relevant mechanisms, food-system implications, and limitations in interpretation. The quantitative values provide context for specific mechanisms and surveillance observations; they should not be treated as pooled effects or global prevalence estimates. Low-concentration selection, gene co-occurrence, altered susceptibility, enhanced horizontal transfer, and crop contamination represent different stages of evidence and therefore cannot be interpreted as equivalent indicators of clinically relevant transmission (2–4, 13).

**Table 1:** Evidence linking environmental pollutant pressures to AMR-relevant processes in food systems. Source: synthesis of the primary studies cited in the table.

Pollutant pressure	Evidence relevant to AMR	Food-system implication	Interpretation caution
Antibiotic and pharmaceutical residues	Selection can occur below clinical MICs; environmental screening concentrations have been proposed (3, 12). Quantitative anchor: the minimum selective concentration ranged from one-quarter to one-two-hundred-and-thirtieth of the susceptible-strain MIC in the tested antibiotic-resistance combinations (3).	Residues in wastewater, manure, livestock, and aquaculture can sustain selective pressure.	A screening concentration is not a universal safe limit or proof of clinical risk.
Metals	ARGs and metal-resistance genes can co-occur on plasmids and in wastewater communities (2, 19, 20). A comparative genomic study examined 2,522 complete bacterial genomes and 4,582 plasmids and demonstrated substantial co-occurrence potential among antibiotic-, biocide-, and metal-resistance determinants (2). These findings support targeted investigation of metal-associated co-selection, but co-occurrence alone does not establish that a measured field concentration caused antibiotic resistance.	Persistent metals may maintain linked determinants in soil, water, feed, and processing waste.	Co-occurrence or correlation does not demonstrate causation at a measured field concentration.
Pesticide formulations	Sublethal herbicide exposure changed antibiotic susceptibility in *E. coli* and *S. enterica*, with direction and magnitude dependent on the combination tested (21). Quantitative anchor: induced MIC changes reached sixfold in the tested herbicide-antibiotic-species combinations (21).	Agricultural co-exposure may alter bacterial responses during production.	Laboratory effects should not be extrapolated to all compounds, residues, or field settings.
Biocides and disinfectants	Food-associated *L. monocytogenes* may carry bcrABC; biocide exposure can select pathways shared with antibiotic resistance (22, 23). Quantitative anchors: 13 of 59 retail-food *L. monocytogenes* isolates were benzalkonium-chloride resistant, with bcrABC in 2 of the 13; all four biocides tested in *Salmonella* selected mutants with decreased antibiotic susceptibility (22, 23).	Repeated sanitation exposure may favor persistent facility strains when cleaning is poorly designed or verified.	Correctly applied disinfection remains essential; evidence does not justify reducing hygiene.
Microplastics	Plastic-associated biofilms can increase plasmid transfer compared with free-living communities (4). Quantitative anchor: plasmid transfer reached approximately one transconjugant per 46 donor cells on microplastics compared with one per 100,000 cells in the surrounding water (4).	Plastics may provide surfaces where bacteria, pollutants, and mobile elements co-occur.	Gene transfer does not establish that a viable foodborne pathogen reaches consumers.
Mixed wastewater and effluents	Wastewater can contain selective chemicals, resistant bacteria, ARGs, and mobile elements together (19). In a controlled lettuce study, secondary-treated irrigation yielded mean *E. coli* abundance of 1.26 ± 0.51 log10 CFU/g and ESBL-producing *E. coli* in 61% of plants at 0.61 ± 0.39 log10 CFU/g; with potable or tertiary-treated water, mean *E. coli* remained below LOQ and ESBL-producing *E. coli* below LOD (13).	Irrigation, aquaculture, and food-processing water may connect environmental reservoirs to food.	Risk depends on treatment performance, commodity, exposure, and organism viability.

AMR, antimicrobial resistance; ARG, antibiotic resistance gene; CFU, colony forming unit; E. coli, *Escherichia coli*; ESBL, extended-spectrum beta-lactamase; L. monocytogenes, *Listeria monocytogenes*; LOD, limit of detection; LOQ, limit of quantification; MIC, minimum inhibitory concentration; S. enterica, *Salmonella enterica*.

Antibiotic residues provide the most direct selective pressure. Experimental work has shown selection of resistant bacteria at concentrations far below the minimum inhibitory concentration (MIC), supporting the concept of a minimum selective concentration (MSC) (3). Predicted no-effect concentrations for resistance selection were consequently proposed for 111 antibiotics as environmental screening values, although these are not universal safety thresholds and remain dependent on compound, microbial community, exposure duration, and matrix (12). Metals can contribute to co-selection when metal- and antibiotic-resistance determinants are genetically linked or when shared physiological responses alter susceptibility. A genomic analysis of 2,522 complete bacterial genomes and 4,582 plasmids found frequent co-occurrence of antibiotic-, biocide-, and metal-resistance genes, while wastewater analysis detected intI1 and ARGs alongside metal-resistance genes including czcA and arsB (2, 19). A regional food-exposure study from Bayelsa State, Nigeria, separately demonstrated measurable dietary exposure to trace-metal mixtures in fish (27). That case is relevant as an example of chemical exposure in a food system, but it does not demonstrate metal-driven AMR; such an inference would require matched concentration-response, microbiological, mobility, and exposure evidence (20).

Evidence for pesticides and biocides is compound- and organism-specific. In *Escherichia coli* and *Salmonella enterica serovar Typhimurium*, sublethal exposure to commercial herbicide formulations changed susceptibility to several antibiotic classes by as much as sixfold, with increases or decreases depending on the herbicide-antibiotic-species combination (21). Benzalkonium-chloride resistance in food-associated *Listeria monocytogenes* has been linked to the bcrABC cassette, and experimental exposure to each of four tested biocides selected *Salmonella* mutants with decreased susceptibility to one or more antibiotics (22, 23). Microplastics can adsorb chemicals and support dense biofilms; in aquatic microcosms, plasmid transfer reached approximately one transconjugant per 46 donor cells on microplastics compared with one per 100,000 cells in the surrounding water (4). These results justify targeted surveillance of real exposure concentrations, mixtures, biofilms, and resistance phenotypes, but they should not be generalized to every pesticide, disinfectant, plastic, organism, or food environment. Correctly designed and verified disinfection remains essential to food safety, while the public-health consequence of enhanced gene transfer remains uncertain unless a viable pathogen, mobile determinant, and plausible exposure pathway are demonstrated (4, 21–23).

### 2.2 Mechanisms that matter clinically

Co-selection includes co-resistance, in which different determinants are genetically linked; cross-resistance, in which one mechanism confers decreased susceptibility to multiple agents; and co-regulation, in which a shared regulatory response affects several stressors. Plasmids, transposons, integrons, and other mobile genetic elements increase concern because they can move determinants between environmental bacteria, commensals, colonizing organisms, and pathogens. Class 1 integrons are genetic platforms rather than resistance genes themselves: intI1 encodes the integrase, while cassette arrays and conserved regions may contain resistance determinants (19, 24). Four analytical states should be kept distinct. Detection of an antimicrobial resistance gene (ARG) establishes sequence presence; evidence of transcription or another functional readout supports expression; antimicrobial susceptibility testing establishes a phenotypic resistance or decreased-susceptibility profile in a cultured organism; and viability testing establishes that the organism is capable of growth or otherwise remains biologically intact. None of these states alone proves clinically relevant transmission. That inference requires a viable organism capable of colonization or disease, a relevant expressed or phenotypic resistance trait, sufficient exposure, and epidemiological or source-attribution evidence linking the environmental or food-system source to people. Infectious dose varies with strain, food matrix, preparation, route, and host susceptibility, as demonstrated in a challenge study involving 111 volunteers exposed to *Campylobacter jejuni* doses from 8 × 10∧2 to 2 × 10∧9 organisms (25). Global stress regulators should also be interpreted cautiously. In 328 natural *E. coli* isolates tested against 15 antibiotics, RpoS level had only a very small overall association with resistance, and its effects on tolerance or persistence to ampicillin, ciprofloxacin, and kanamycin were not statistically significant (28). Thus, stress-response markers cannot substitute for phenotypic resistance, organism viability, genomic context, and exposure evidence.

## 3 From environmental selection to foodborne public-health risk

### 3.1 Evidence hierarchy and clinical relevance

The convergence framework prevents two common errors. The first is treating any multidrug-resistant environmental isolate as evidence of human pollution, despite the intrinsic resistome documented in bacteria from isolated environments (5). The second is treating an ARG detected by metagenomics as evidence that a viable foodborne pathogen carries and expresses it. A practical evidence hierarchy should distinguish intrinsic resistance, acquired but apparently non-mobile resistance, mobile clinically relevant resistance, and viable pathogenic or opportunistic organisms carrying mobile resistance. Priority increases when the determinant affects treatment, is located on a transmissible element, occurs in a viable organism, and is connected to a frequently consumed or minimally processed food. The RpoS findings reinforce this caution: even a major global stress regulator had only a very small overall relationship with resistance across 328 natural *E. coli* isolates, so single genes or stress markers should not be used as surrogates for clinical threat (Valencia et al., 2024). The hierarchy is therefore a ranking tool for surveillance and intervention, not a claim that every environmental resistance signal is harmless.

### 3.2 Wastewater irrigation and post-farm exposure

Wastewater illustrates why context matters and why quantitative results should be treated as setting-specific case examples. In a controlled lettuce study*, E. coli* and ESBL-producing *E. coli* occurred in all 18 secondary-treated irrigation-water samples, at mean concentrations of 4.59 ± 0.44 and 2.61 ± 0.33 log10 CFU/100 mL, respectively (13). On lettuce irrigated with secondary-treated water, *E. coli* was detected in 94% of samples at 1.26 ± 0.51 log10 CFU/g, while ESBL-producing *E. coli* occurred in 61% at 0.61 ± 0.39 log10 CFU/g. With potable or tertiary-treated irrigation, *E. coli* was still detected in 33.3% of lettuce samples, but mean abundance remained below the limit of quantification (LOQ); ESBL-producing *E. coli* remained below the limit of detection (LOD) (13). A separate romaine-lettuce study found 140 selected *E. coli* isolates across wastewater, foliage, soil, and leachate, of which 81% resisted ampicillin and 34% resisted cephalothin (14). These studies illustrate crop-production pathways, not universal wastewater effects, because outcomes vary with treatment performance, crop, irrigation method, soil, climate, baseline microbiota, and time to harvest. Post-farm pathways are likewise context dependent. A meta-analysis from Bayelsa State, Nigeria, identified multiple microbial genera in ready-to-eat foods (15). These findings provide a regional case example supporting inclusion of markets, vendors, processing environments, and households and should not be interpreted as a global prevalence estimate. By contrast, genomic comparison in the United Kingdom and source-attribution modeling in the Netherlands show that the proportional contribution of livestock, food, environmental, and human reservoirs can differ markedly by setting (16, 17). Implementation of the WHO Tricycle protocol in Madagascar further demonstrates the value of harmonized human-animal-environment sampling for testing intersectoral circulation rather than assuming it (29). Together, these examples from Africa and Europe reinforce the central point that pathway strength and source contribution must be measured locally while using harmonized surveillance principles.

## 4 Discussion and future directions

### 4.1 Surveillance designed for causal inference

A One Health hotspot should not be defined solely by the highest pollutant concentration or ARG abundance. Existing frameworks provide the essential architecture for integrated surveillance. WHO/AGISAR emphasizes coordinated surveillance across human, animal, and food sectors; Tricycle operationalises comparable ESBL-producing *E. coli* surveillance across human, food-chain, and environmental compartments; UNEP identifies environmental pollution sources and prevention priorities; and the 2026 Quadripartite guidance emphasizes objective-driven design, harmonized sampling and metadata, laboratory and epidemiological capacity, integrated analysis, data sharing, and communication (7–10). The Global Action Plan on AMR 2026–2036 places these activities within a broader One Health implementation and accountability agenda (11). Within this architecture, pollutant-associated AMR can be evaluated through a source-pathway-receptor chain linking measured pollutant mixtures to viable resistant organisms, resistance phenotype, ARG abundance and functional context, mobile-element linkage, food contamination, consumption, and clinical consequence. The analytical package should be matched to the surveillance objective and available capacity. Culture and standardized antimicrobial-susceptibility testing establish viability and phenotype; chemical analysis characterizes selective pressure; whole-genome sequencing of representative isolates supports strain and resistance-context analysis; targeted metagenomics or qPCR can quantify selected resistance determinants; and long-read sequencing can resolve plasmids, transposons, or integrons when mobility is central to attribution. Exposure assessment should consider commodity, serving size, consumption frequency, preparation, time-temperature history, and relevant host factors. Source-attribution studies illustrate the importance of this integration. In the United Kingdom, only two of 1,517 human bloodstream *E. coli* isolates were within 15 core-genome SNPs of the 431 livestock/meat isolates examined (16). In the Netherlands, human-to-human transmission accounted for the largest estimated contribution to community ESBL/pAmpC carriage, while food and other non-human pathways made smaller but measurable contributions (17). These findings do not diminish food or environmental reservoirs; rather, they demonstrate that their contribution is context-specific and requires direct testing. WGS networks strengthen food-pathogen traceback when genomic similarity is interpreted alongside time, place, exposure, mobile-element, and supply-chain information (18).

### 4.2 Testable propositions and action

Four propositions follow from the evidence and warrant prospective evaluation. Typically, pollutant-mixture measures combined with mobile-element information may identify clinically relevant AMR enrichment more effectively than a single pollutant concentration. Also, public-health risk is expected to be greatest where viable organisms, clinically relevant resistance, and frequently consumed or minimally processed foods overlap. Additionally, interventions that reduce both selective chemicals and viable resistant bacteria may provide more durable control than approaches evaluated only through total bacterial counts. Moreover, strain or plasmid sharing across environmental, food, and clinical compartments is likely to provide stronger evidence of transmission than resistome similarity alone. These propositions can be examined through multisite longitudinal studies, repeated co-located sampling, natural experiments around treatment upgrades, controlled production or processing studies, and linkage to food-consumption and clinical data. The evidence supporting low-concentration selection, wastewater-treatment effects, genomic comparison, and source attribution provides a basis for these hypotheses while also showing why their magnitude cannot be assumed across settings (3, 13, 16, 17).

Policy responses should distinguish established control practices from pollutant-specific interventions that still require evaluation. Antimicrobial stewardship, diagnostic microbiology, food-hygiene controls, fit-for-purpose wastewater management, and coordinated One Health surveillance are supported by established public-health and food-safety practice. In contrast, the benefit of targeting a particular pollutant mixture or predicting a specific change in ARG mobility is likely to depend on the local source, concentration, microbial community, treatment system, commodity, and exposure pathway. Evaluation should therefore preserve distinctions among detection, quantification, viability, phenotype, mobility, exposure, and clinical consequence. In wastewater-irrigated lettuce, for example, detection of *E. coli* below the limit of quantification was analytically different from non-detection of ESBL-producing *E. coli* below the stated limit of detection (13). Such distinctions are necessary if environmental surveillance is to support proportionate and evidence-based intervention.

## 5 Conclusion

Environmental pollutants can modify the ecology and transmission potential of AMR across food systems, but pollutant detection, ARG presence, multidrug resistance, or genomic similarity alone does not establish clinically relevant human transmission. The strongest public-health concern arises where selective pressure coincides with viable pathogens or colonizing organisms, clinically relevant phenotypic or expressed resistance, mobile determinants, and meaningful human exposure. Existing One Health frameworks provide the architecture for integrated cross-sector surveillance; a pollutant-explicit perspective adds value by making the transitions from environmental exposure to biological response, viability, mobility, food-system exposure, and source attribution explicit. Current evidence supports biological plausibility for selection below the MIC, co-selection, altered susceptibility, and enhanced gene transfer, while wastewater and source-attribution studies demonstrate that the strength of transmission pathways is highly context-dependent. Future surveillance should therefore prioritize co-located chemical and microbiological measurements, genomic context, exposure assessment, and source attribution rather than gene abundance alone. The most useful interventions will be those that can demonstrate sustained reductions in selective pressures, viable resistant organisms, food contamination, or clinically relevant exposure. Framing environmental pollutants as modifiers of AMR risk provides a cautious basis for action while preserving the evidential distinction between environmental occurrence and human health consequence.

## Data Availability

The original contributions presented in the study are included in the article/supplementary material, further inquiries can be directed to the corresponding author.
